# Individual DOI minting for Open Repository: a script for creating a DOI on demand for a DSpace repository

**DOI:** 10.5195/jmla.2025.2076

**Published:** 2025-01-14

**Authors:** Tess Grynoch, Lisa A. Palmer

**Affiliations:** 1 Tess.Grynoch@umassmed.edu, Research Data & Scholarly Communications Librarian, Lamar Soutter Library, University of Massachusetts Chan Medical School, Worcester, MA; 2 Lisa.Palmer@umassmed.edu, Institutional Repository Librarian, Lamar Soutter Library, University of Massachusetts Chan Medical School, Worcester, MA

**Keywords:** Institutional Repositories, DSpace, Open Repositories, DataCite, Python

## Abstract

Digital Object Identifiers (DOIs) are a key persistent identifier in the publishing landscape to ensure the discoverability and citation of research products. Minting DOIs can be a time-consuming task for repository librarians. This process can be automated since the metadata for DOIs is already in the repository record and DataCite, a DOI minting organization, and Open Repository, a DSpace repository platform, both have application programming interfaces (APIs). Existing software enables bulk DOI minting. However, the institutional repository at UMass Chan Medical School contains a mixture of original materials that need DOIs (dissertations, reports, data, etc.) and previously published materials that already have DOIs such as journal articles.

An institutional repository librarian and her librarian colleague with Python experience embarked on a paired programming project to create a script to mint DOIs on demand in DataCite for individual items in the institution's Open Repository instance. The pair met for one hour each week to develop and test the script using combined skills in institutional repositories, metadata, DOI minting, coding in Python, APIs, and data cleaning. The project was a great learning opportunity for both librarians to improve their Python coding skills. The new script makes the DOI minting process more efficient, enhances metadata in DataCite, and improves accuracy. Future script enhancements such as automatically updating repository metadata with the new DOI are planned after the repository upgrade to DSpace 7.

## BACKGROUND

The eScholarship@UMassChan institutional repository [1] is a digital archive and dissemination platform for the scholarship of students, faculty, and staff at UMass Chan Medical School in Worcester, Massachusetts. eScholarship@UMassChan utilizes Open Repository version 5.7, a hosted software platform from Atmire built on DSpace software [2]. The repository contains the full text of previously published items, such as journal articles, along with original materials, including theses, dissertations, posters, reports, and datasets.

For all original materials submitted, repository staff creates a Digital Object Identifier (DOI) via DataCite [3], a DOI minting organization. This is a crucial service, as DOIs are a key persistent identifier in the publishing landscape to ensure discoverability and citation of research products. DSpace repositories can mint DOIs automatically for all items, but this feature is not suitable for repositories that include published materials which already have DOIs. Thus, repository staff minted DOIs by entering metadata for each original resource into an online form, a time-consuming task open to error.

Knowing this process could be automated, the authors, a repository librarian and a data librarian colleague with coding experience, embarked on a paired programming project to create a Python script to mint DOIs on demand in DataCite for individual items in eScholarship@UMassChan.

## THE PAIRED PROGRAMMING PROJECT

The repository librarian and data librarian met for one hour each week starting in July 2023 to develop the script. This approach took advantage of the librarians' combined skills in institutional repositories, metadata, DOI minting, coding in Python, APIs, and data cleaning. An important step was to create a crosswalk to map metadata values for document types from DSpace to DataCite. The project team also knew that the repository would be upgraded to DSpace 7 in 2024 and have a new API, leading them to only use the Open Repository API to download but not edit repository metadata. Another decision point was how to handle items with multiple authors with ORCID IDs, as repository metadata does not link authors with their ORCID. The project team decided that the script would utilize the ORCID field if the item had one author but not for multiple authors. So, items with multiple ORCID IDs still need to have IDs added to DataCite manually.

The script was successfully used to upload repository metadata to the DataCite test server in November 2023 and the production version of the script was created and tested in December 2023. A de-identified version of the script was published on GitHub in January 2024 and can be modified for use in other DSpace 5.7 repositories [4].

## IMPACT AND FUTURE DIRECTIONS

The new script allows repository staff to mint DOIs more efficiently (3–13 minutes faster) with improved metadata and fewer human errors. Library users and the institution benefit because the institutional repository librarian has more time to enhance and add content to the repository. The project team also gained new skills that can be applied to additional opportunities to improve library processes and services to users. After the anticipated DSpace 7 upgrade, the project team plans to update the script using the new API. The project team is also monitoring DSpace/ORCID integration efforts that could improve the process [5].
